# Rapidly reversible paraplegia after coarctation repair in an infant: Potential reperfusion injury relieved by cerebrospinal fluid drainage

**DOI:** 10.1016/j.xjtc.2026.102318

**Published:** 2026-03-17

**Authors:** Myriam Addi, Jonathan Au Duong, Gérald Chausseray, Julie Gobin, Yves Dulac, Lionel Berthomieu, Najeebullah Bina, Bertrand Marcheix

**Affiliations:** a Department of Cardiac Surgery, Hôpital des Enfants, Toulouse, France; b Department of Pediatric and Neonatal Medical and Surgical Intensive Care, Hôpital des Enfants, Toulouse, France; c Department of Pediatric Cardiology, Hôpital des Enfants, Toulouse, France

**Figure:**
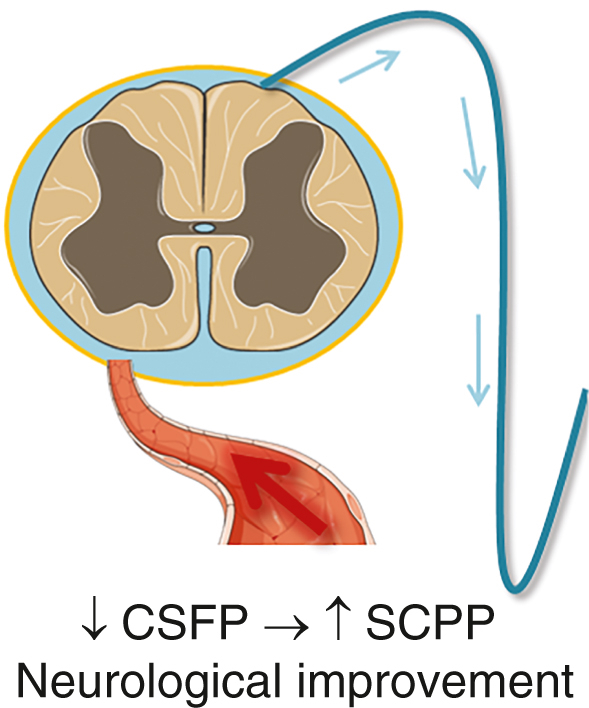
Lowering CSFP increases spinal cord perfusion pressure and improves neurologic recovery.

**Central Message**

Spinal cord ischemia after coarctation repair can be reversible; CSF drainage restores perfusion and enables rapid neurologic recovery.

Spinal cord ischemia resulting in paraplegia is a feared but rare complication after surgical repair of aortic coarctation, with an incidence of less than 1% reported in pediatric series.^1^ Anatomical factors influencing spinal cord collateral circulation—including intercostal and vertebral arteries—may modulate individual susceptibility, although this is rarely documented in young children. Early recognition and prompt intervention are critical, because cerebrospinal fluid (CSF) drainage improves spinal cord perfusion pressure and prevents neurologic injury in adults who undergo descending aortic surgery.^2^ Spinal cord ischemia after coarctation repair in infants is exceptional, and the role of CSF drainage in this setting has not been reported. We report the case of a 20-month-old infant who developed acute paraplegia immediately after coarctation repair, with complete recovery after CSF drainage.

## Case Report

A 20-month-old boy (weight 8 kg), was referred for surgical correction of aortic coarctation. Preoperative echocardiography showed a restrictive perimembranous ventricular septal defect, a small ductus arteriosus, and a severe isthmic coarctation with preserved biventricular function.

**Figure 1:**
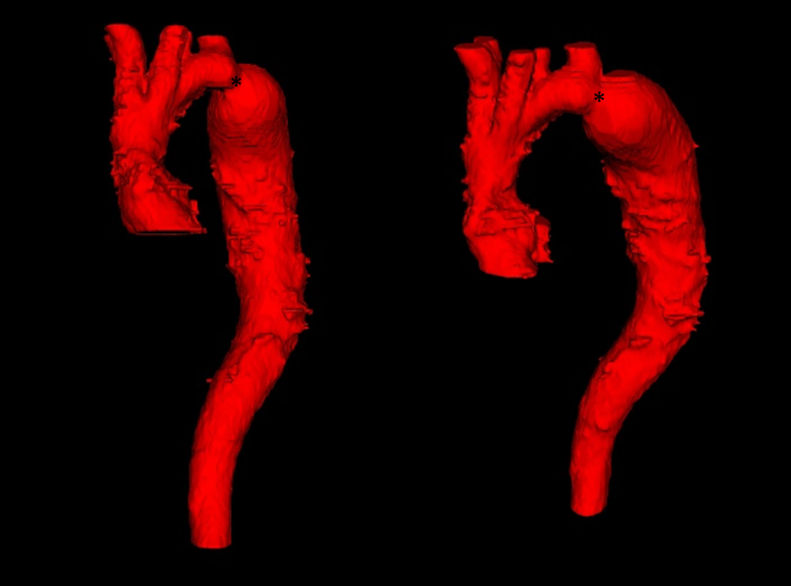
Three-dimensional reconstruction of the thoracic aorta showing severe aortic coarctation (∗) with post-stenotic aneurysmal dilatation.

Preoperative computed tomography angiography (Figure 1) confirmed an isthmic coarctation with a poststenotic aneurysmal dilatation. The scan also demonstrated an isolated origin of the left vertebral artery. Imaging assessment of intercostal arteries was limited, but intraoperative findings suggested sparse collateralization.

The surgical repair was performed via a left posterolateral thoracotomy in the fourth intercostal space with the patient under general anesthesia and with invasive monitoring. Distal perfusion was monitored using a femoral line. During aortic crossclamping, mean femoral arterial pressure was maintained at approximately 50 mm Hg. The procedure was performed under normothermia.

Given the presence of a significant poststenotic aneurysmal segment with marked caliber mismatch between the aortic arch and the descending aorta, a Waldhausen-type repair was planned. This consisted of an extended end-to-end anastomosis with sacrifice of the left subclavian artery to achieve a tension-free reconstruction.

The ductus arteriosus was ligated. After test clamping, a single continuous aortic crossclamp was applied for 30 minutes, without intermediate unclamping. The repair was hemostatic, with no residual gradient at declamping. Near-infrared spectroscopy showed stable muscular but decreased renal values. The patient was transferred intubated to the intensive care unit requiring low-dose inotropic support to optimize cardiac output. A concomitant hypertensive tendency was managed with low-dose intravenous urapidil.

In the immediate postoperative period, the patient was awake and responsive. Upper-limb motor function was preserved, whereas there was a complete absence of movement in both lower limbs. Residual neuromuscular blockade was excluded. The neurologic presentation was consistent with early phase of spinal shock secondary to acute spinal cord ischemia, based on flaccid paraplegia, absent deep tendon reflexes, and anal sphincter relaxation. However, precise segmental localization was not possible at that stage because of the acute phase of spinal shock and the absence of immediate spinal imaging.

**Figure E1:**
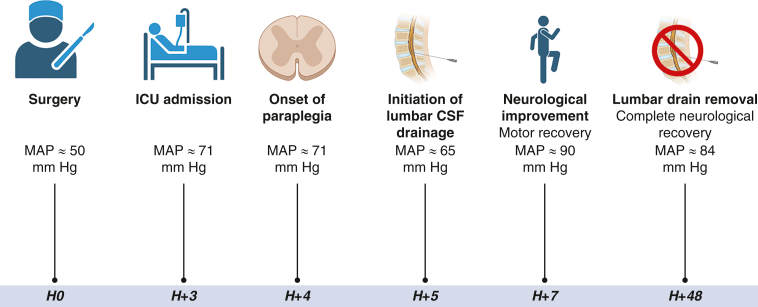
Timeline of perioperative events and neurologic outcome. Schematic representation of surgical timing, onset of neurologic deterioration, initiation of lumbar cerebrospinal fluid (CSF) drainage, and subsequent neurologic recovery. Despite apparently acceptable mean arterial pressure (MAP) values during and after aortic crossclamping, rapid motor recovery after CSF drainage supports a transient spinal cord hypoperfusion mechanism. *ICU*, Intensive care unit.

After control of coagulation parameters, lumbar CSF drainage was initiated as an emergency therapeutic measure using a neurosurgical drainage system. Management included elevated mean arterial pressure, corticosteroids, and CSF drainage. Given the patient's age and weight, no predefined CSF pressure target was applied. Drainage was guided by neurologic status and hemodynamics (Appendix E1). Motor function began to recover by midnight on postoperative day 0. By postoperative day 2, deep tendon reflexes and antigravity strength were restored, allowing drain removal (total drainage: 358 mL). The sequence of perioperative events and neurologic findings is summarized in Figure E1.

Postoperative complications included chylothorax, paralytic ileus, and bacteremia, all of which were treated successfully. Follow-up echocardiography confirmed excellent surgical results.

Magnetic resonance imaging of the spine performed 2 weeks later was unremarkable. Neurologic examination on discharge and at follow-up showed complete recovery with normal gait and only mild brisk reflexes.

Written informed consent for publication was obtained from the patient's legal guardians, in accordance with institutional policy. Institutional review board approval was not required.

## Discussion

Spinal cord perfusion depends on a complex collateral network involving the anterior and posterior spinal arteries and segmental feeders.^3^^,^^E1^ The artery of Adamkiewicz supplies the anterior spinal cord. The anterior horns, responsible for motor function, are therefore particularly vulnerable to ischemia.

During aortic surgery, interruption of intercostal or subclavian flow, prolonged crossclamping, or hypotension can critically reduce spinal perfusion pressure. The spinal cord's tolerance to ischemia is limited, with irreversible injury after 5 to 8 minutes of perfusion arrest.^4^^,^^E2^

**Figure 2:**
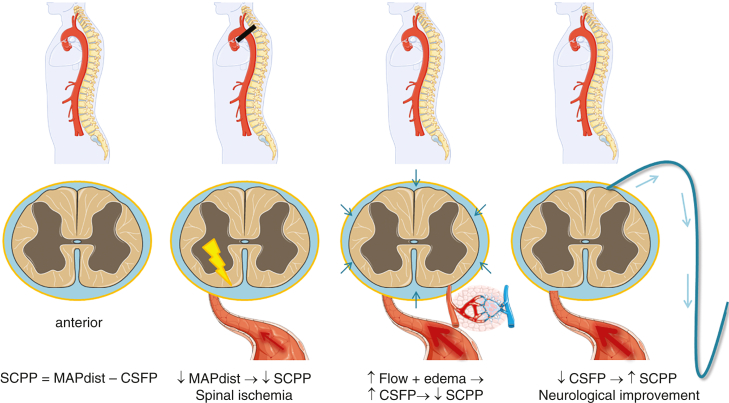
Schematic representation of spinal perfusion and the role of cerebrospinal fluid (CSF) drainage in improving spinal cord perfusion pressure (SCPP = distal mean arterial pressure [MAPdist]– cerebrospinal fluid pressure [CSFP]). From *left to right*: normal situation, aortic clamping, declamping and reperfusion, and after CSF drainage. Some elements of this figure were adapted from Servier Medical Art, licensed under a CC BY 3.0 license.

CSF drainage increases spinal cord perfusion pressure by lowering intrathecal pressure (Figure 2): spinal cord perfusion pressure = distal mean arterial pressure – cerebrospinal fluid pressure. This strategy is a cornerstone of spinal protection in thoracoabdominal aortic surgery and may be lifesaving when postoperative neurologic deficits occur.^E3^

In our case, intraoperative inspection suggested markedly limited intercostal collateralization, which could have reduced the spinal cord's tolerance to decreased distal perfusion during aortic crossclamp. Despite an apparently acceptable distal perfusion pressure during crossclamping, transient spinal cord hypoperfusion likely occurred.

Preoperative computed tomography remains essential to assess aortic anatomy and collateral circulation. Although this case does not justify changes in imaging or surgical strategy, careful assessment of distal perfusion may help identify rare situations requiring alternative protective strategies.

The abrupt onset of neurologic deficits in the immediate postoperative period strongly suggested a vascular mechanism rather than a compressive etiology, further supporting the decision to prioritize emergent therapeutic intervention. The rapid and complete neurologic recovery after CSF drainage supports a diagnosis of transient spinal cord hypoperfusion rather than structural infarction. Such reversible postoperative paraplegia is rare in infants and highlights the importance of early recognition and management. Although endovascular stenting is increasingly used in older children and adults, surgical repair remains the treatment of choice in neonates and infants.^5^^,^^E4^

## Conclusions

This case illustrates that acute paraplegia after coarctation repair may be reversible when promptly treated. CSF drainage should be considered as an emergency measure in any postoperative neurologic deficit suggestive of spinal ischemia, even in infants.

## Conflict of Interest Statement

The authors reported no conflicts of interest.

The *Journal* policy requires editors and reviewers to disclose conflicts of interest and to decline handling or reviewing manuscripts for which they may have a conflict of interest. The editors and reviewers of this article have no conflicts of interest.

## Appendix E1

### Lumbar Cerebrospinal Fluid (CSF) Drainage Protocol used in this infant

#### Indication

Emergency treatment of acute postoperative paraplegia after aortic coarctation repair is highly suggestive of spinal cord hypoperfusion.

#### Technique

Lumbar cerebrospinal fluid drainage was initiated in the intensive care unit using a pediatric neurosurgical drainage system after correction of coagulation parameters.

#### Drainage strategy

Given the patient's age (20 months) and weight (8 kg), no predefined CSF pressure target was applied. Drainage was performed cautiously and guided primarily by neurologic examination and overall hemodynamic tolerance rather than pressure measurements.

CSF was drained intermittently to avoid excessive removal with a maximum drainage rate limited to 10 mL per hour, as agreed with the neurosurgical team, in order to minimize the risk of overdrainage-related complications. Mean arterial pressure was maintained at elevated levels to optimize spinal cord perfusion.

#### Monitoring

Continuous hemodynamic monitoring and frequent neurologic assessments were performed. No signs of overdrainage or procedure-related complications were observed.

#### Duration and volume

A total of 358 mL of CSF was drained over 48 hours. Neurologic recovery began within hours of drainage initiation, with progressive return of lower-limb motor function and reflexes.

#### Termination

The drain was removed on postoperative day 2 after neurological recovery.
